# The effects of CYP450 inhibition on cerebrovascular control during rest and mild hypovolemia: An exploratory study in young, healthy adults

**DOI:** 10.14814/phy2.70712

**Published:** 2025-12-19

**Authors:** Jacob E. Matney, Alexander A. Buelow, Chris Mixon, Sarah M. Skillett, John D. Ashley, Jiwon Song, Amir Akbari Fakhrabadi, Paige Pointer, Justin D. Sprick, Christopher D. Black, Hugo M. Pereira, J. Mikhail Kellawan

**Affiliations:** ^1^ Human Circulation Research Laboratory, Department of Health and Exercise Science University of Oklahoma Norman Oklahoma USA; ^2^ Institute for Exercise and Environmental Medicine, Texas Health Presbyterian Hospital Dallas Dallas Texas USA; ^3^ Department of Neurology University of Texas Southwestern Medical Center Dallas Texas USA; ^4^ Department of Kinesiology, Health Promotion, and Recreation University of North Texas Denton Texas USA; ^5^ Sensory and Muscle Function Research Laboratory, Department of Health and Exercise Science University of Oklahoma Norman Oklahoma USA; ^6^ Human Movement and Neurophysiology Research Laboratory, Department of Health and Exercise Science University of Oklahoma Norman Oklahoma USA

**Keywords:** cerebral autoregulation, cerebral hemodynamics, critical closing pressure, CYP450, epoxyeicosatrienoic acid, hydroxyeicosatetraenoic acid, lower body negative pressure, resistance area product

## Abstract

Cerebrovascular CYP450 is underexplored in humans. Twenty‐three participants (12 females) completed familiarization and two experimental visits utilizing double‐blind, randomized, crossover design. Participants ingested fluconazole (150 mg; FLZ, CYP450 inhibitor) or placebo (PLC, microcrystalline cellulose) followed by 120 min of supine rest. Five minutes of middle cerebral artery velocity (MCAv) and mean arterial pressure (MAP) were collected during rest (PLC/FLZ_BASE_) and lower body negative pressure (LBNP; −20 mmHg; PLC/FLZ_LBNP_). There was no interaction for MCAv, cerebrovascular conductance index (CVCi), resistance area product (RAP), critical closing pressure (CrCP), or transfer function variables. Bonferroni tests revealed only FLZ attenuated MCAv (FLZ_BASE_ 75.2 ± 11.3 cm•s^−1^ vs. FLZ_LBNP_ 70.6 ± 11.2 cm•s^−1^, *p* = 0.015, *d* = 0.713) and CVCi (FLZ_BASE_ 0.76 ± 0.12 cm•s^−1^•mmHg^−1^ vs. FLZ_LBNP_ 0.71 ± 0.11 cm•s^−1^•mmHg^−1^, *p =* 0.003, *d* = 0.865) during LBNP. LBNP lowered CrCP (PLC_BASE_ 29.7 ± 9.3 mmHg vs. PLC_LBNP_ 25.1 ± 11.3 mmHg, *p* = 0.002, *d =* 0.940) within PLC. RAP increased during LBNP (PLC_BASE_ 0.96 ± 0.29 vs. PLC_LBNP_ 1.08 ± 0.32, *p* = < 0.001, *d* = 1.28; FLZ_BASE_ 0.98 ± 0.24 vs. FLZ_LBNP_ 1.07 ± 0.23, *p* = < 0.001, *d* = 1.10). These data suggest a participatory, nonobligatory role of CYP450 in human cerebrovascular control.

## 1 INTRODUCTION

Due to the neuron's inability to store adequate nutrients, the brain demands an uninterrupted supply of nutrient‐rich blood to promote proper health and function. In fact, the brain requires ~15% of resting cardiac output, or roughly 20% of total body oxygen consumption, to maintain proper function at rest despite only representing ~2% of the body's total mass (Clarke & Sokoloff, 1999). As a result, the cerebral vasculature is equipped with numerous mechanisms to ensure continuous blood flow to the brain regardless of challenges in systemic blood pressure, neuronal activation, and metabolic disruptions across the body. Cerebral hemodynamic control—including cerebral autoregulation (CA), the intrinsic ability of the cerebrovasculature to stabilize cerebral blood flow during fluctuations in arterial blood pressure—has been shown to rely on myogenic, metabolic, neurogenic, and endothelium‐dependent factors to regulate cerebral blood flow (Aaslid et al., 1989; Claassen et al., 2021; Hamner & Tan, 2014; Shoemaker et al., 2021; White et al., 1998). However, the exact pathways utilized by the brain's vasculature to maintain persistent blood flow, nor the redundancy of each factor, are not fully understood. Research suggests that impaired cerebrovascular control is linked to increased stroke incidence, poorer post‐stroke outcomes, as well as increased neurodegenerative disease progression (Santos et al., 2022; Taylor et al., 2024). Not only is the prevalence of stroke rising, but so are survival rates (Pu et al., 2023). Thus, there is an urgent need to better understand cerebral blood flow and the intrinsic mechanisms of hemodynamic control in the human cerebrovasculature in health and disease to better prevent the formation of and enhance the recovery from cerebrovascular disease such as stroke.

The production of eicosanoids through cytochrome P450 enzymes, particularly epoxyeicosatrienoic (EETs) and hydroxyeicosatetraenoic (HETEs) acids, has long been of clinical interest due to their relevance to cardiovascular, neurological, and inflammatory diseases and have proven important for cerebral blood flow regulation (Huang et al., 2016; Roman, 2002). Harder and colleagues have demonstrated that 20‐HETE production resulted in vasoconstriction of pial arteries in cats in vitro (Harder et al., 1994). Gebremedhin et al. (2000) demonstrated that the HETE metabolites influence CA responses to hypertensive stimuli in rats, where inhibition of HETE production limited CA response assessed through an autoregulatory index. Amruthesh et al. (1992) found that EETs were produced by the brain in rats and that brain‐synthesized EETs caused dilation of the cerebral microvasculature in rabbits. Importantly, other evidence shows that excessive HETEs production in the brain's microvasculature has been linked to the development of cerebral vasospasms, increased severity of stroke infarct volume, and the degradation of post‐stroke cerebrovascular control (Huang et al., 2016). Conversely, studies examining EETs demonstrate that increased EET concentrations protect the cerebrovasculature from the negative aftermath of stroke through vasodilation and angiogenesis, reducing stroke infarct size in animals (Dorrance et al., 2005; Simpkins et al., 2009). Clearly, EETs and HETEs play an important role in hemodynamic regulation within the brain in animal models, as well as a predictor of post‐stroke health outcomes; however, very few have manipulated CYP450 activity directly.

Animal studies examining the effects of CYP450 manipulation, rather than effects of the enzyme's products, are few. In human tissue, research ex vivo has demonstrated that endothelial cells cultured from the cerebral microvasculature express numerous CYP450 isoforms (Ghosh et al., 2010). Moreso, cultured human endothelial cells from cerebral microvessels who experience epilepsy showed an over expression of CYP450 enzymes (Ghosh et al., 2010). Evidence has shown that subdural injection of miconazole (a CYP450‐antagonist) resulted in large reductions of blood flow within the cerebral cortex of mice (Alkayed et al., 1996, 1997). Moreso, this inhibition was found to predominantly inhibit astrocytic CYP450 activity, thus affecting the metabolic environment of the microvasculature (Alkayed et al., 1996, 1997). In transgenic mice, overexpression of CYP450 in the middle cerebral artery vascular endothelium resulted in increased cortical perfusion following middle cerebral artery occlusion in males but not females (Jia et al., 2016). Together, these models demonstrate a crucial importance of CYP450 activity on cerebrovascular control in animals and possibly in humans—especially during states where HETE and EET production are imbalanced, such as stroke or epilepsy. Further, research in the periphery have demonstrated that CYP450 may be a redundant mechanism to nitric oxide (NO), where it has been shown that those with endothelial dysfunction and NO inhibition rely more heavily on CYP450 activity to regulate flow in the absence of NO (Ozkor et al., 2010). However, whether CYP450 contributes similarly to cerebrovascular control in health and disease in humans in vivo remains unknown. Moreso, whether there are sex‐related differences between these effects are also unclear.

CYP450's control on the vasculature appears to be predominantly formed within the microvascular environment—whether by the expression of CYP450 epoxygenase in the endothelium of small diameter vessels or by affecting astrocytic, metabolic‐mediated signaling to the microvasculature. Because of this, exploration of all branches of the cerebral vascular tree is crucial to properly understanding CYP450's role in vivo. Cerebral critical closing pressure (CrCP) and resistance area product (RAP) have been shown to strongly represent changes in metabolic and myogenic activity in the vasculature, respectively (Beishon et al., 2018; Ince et al., 2023; Panerai, 2003). In line with this relationship, it is also believed that these metrics represent changes in capillary and arteriole tone; it is well accepted that the metabolic activity of vessels in the brain predominantly occurs at the level of the capillaries due to glial cell activity and the blood‐brain barrier, while myogenic tone occurs within the arterioles (Ince et al., 2023). Evidence modeling collapsible vessels demonstrated CrCP as a crucial point where resistance through an artery is determined either by the sum resistance of arterioles and capillaries (above CrCP) or by just arteriole resistance alone (below CrCP) (Permutt & Riley, 1963). Thus, the use of CrCP and RAP can give two major benefits to researchers: (1) provide a more accurate assessment of vascular activity at different levels of the vascular tree in the brain where CrCP is more likely to be reached, and (2) reflect myogenic (RAP) and metabolic (CrCP)‐mediated changes in the vasculature.

Therefore, the purpose of this study was to investigate (1) whether static and dynamic cerebrovascular control is impaired following CYP450 inhibition at rest and during mild simulated hypovolemia in healthy, young adults and (2) whether there are potential sex differences in these responses. We hypothesize that resting hemodynamic control, as well as responses (both static and dynamic) to mild hypovolemia, will be worsened following CYP450 inhibition—where females will display worse cerebrovascular impairment than males.

## 2 METHODS

### 2.1 Ethical approval

All study protocols were approved by the University of Oklahoma Health Science Institutional Review Board (No. 14056). Participants were given verbal instructions of all procedures, purpose, and risks involved before providing written consent. The investigation conformed to the standards by the Declaration of Helsinki and was registered on ClinicalTrials.gov (NCT05176379).

### 2.2 Study participants

Twenty‐three young (21.6 ± 2.9 years, 12 females), healthy adults participated in the study. All participants were 18–30 years of age, nonobese, and free of cardiovascular, metabolic, respiratory, neurological, or psychological ailments as assessed by a custom medical history questionnaire. All participants were non‐nicotine users for at least 6 months prior to study enrollment and were not taking any prescription medications throughout the study except for birth control. All female participants were nonpregnant and had a regular menstrual cycle and/or were taking birth control that allowed for regulated menstruation. Non‐pregnancy was confirmed by a commercially available pregnancy test administered prior to each visit by research staff (AcuMed, Houston, TX, USA). All females were tested within the first 1–5 days of their self‐reported menstrual cycle or during the placebo phase of birth control. All participants abstained from caffeine, vigorous exercise, alcohol consumption, and supplements for at least 12 h prior to each visit. Additionally, participants were instructed to attend all visits fasted for a minimum of 8 h and not to consume any non‐steroidal anti‐inflammatory drugs (NSAIDs) within 24 h prior to each visit.

### 2.3 Experimental design

This study employed a randomized, double‐blind, placebo‐controlled, repeated measures crossover design. All participants completed three study visits. Visit 1 consisted of informed consent, eligibility determination, as well as the familiarization of protocol equipment and procedures. Visits 2 and 3 were separated by ≥72 h and were identical in design except for the ingestion of either fluconazole (150 mg tablet; NDC 62559‐992‐12) or placebo (250 mg microcrystalline cellulose tablet; Zeebo Effect, Burlington VT, USA). This dose of fluconazole (150 mg) has been shown to be an effective dose to adequately inhibit CYP450 in previous studies without affecting resting central cardiac responses (Buelow et al., 2025; Petterson et al., 2021). Due to the half‐life of fluconazole (25–27 h), 72 h between visits was chosen to ensure adequate washout between experiments (Grant & Clissold, 1990). Experimental visits were completed in a dim, quiet laboratory with an ambient temperature of 18°C–21°C.

#### 2.3.1 Screening and familiarization visit

Visit 1 consisted of informed consent, Health Insurance Portability and Accountability Act (HIPAA) authorization, study eligibility determination through medical questionnaire and anthropometric measurements, and middle cerebral artery depth and position identification. Middle cerebral artery velocity (MCAv) signal location, depth, and gain were oriented to allow for optimal vessel insonation and were recorded to ensure consistency between study visits (Aaslid et al., 1982; Willie et al., 2011). Participants were then familiarized with experimental measurements and procedures. For lower‐body negative pressure (LBNP), participants were sealed into the air‐tight chamber at the iliac crest and familiarized with the sensation of −20 mmHg pressure, as well as the sealing process. All females underwent a urine‐based pregnancy test prior to any data collection and were excluded if pregnant.

#### 2.3.2 Experimental visits 2 and 3

Prior to each experiment, participants were refamiliarized with all experimental protocols and equipment. All participants gave verbal confirmation of their adherence to pre‐study visit guidelines, and all females performed a pregnancy test to confirm non‐pregnancy. Participants then randomly ingested either a single dose of fluconazole (FLZ) or placebo (PLC). Following ingestion, participants laid in a supine position for 60 minutes to allow for the absorption of the treatment. 60 min post‐ingestion, each participant underwent instrumentation and was then sealed into the LBNP chamber in a supine position with arms positioned at heart level. Following an additional 60 min (totaling 120 min post‐ingestion, with at least 15 min of undisturbed rest prior to data collection), each experimental protocol began. 120 min of post‐ingestion absorption was allowed to ensure peak plasma concentrations of the treatment were reached before each experiment began (Grant & Clissold, 1990). Baseline measurements were collected for 5 min followed by 5 min of LBNP at −20 mmHg, followed by, 5 min of recovery data were recorded.

### 2.4 Materials and equipment

All equipment was calibrated in accordance with manufacturer specifications prior to each study visit.

Heart rate (HR) was continuously collected using a II‐lead electrocardiogram (ECG) vest (Equivital Lifemonitor System, ADInstruments, Colorado Springs, CO, USA) at a rate of 256 Hz on a beat‐to‐beat basis. Mean arterial pressure (MAP), stroke volume (SV), cardiac output (Q), and total peripheral resistance (TPR) were continuously collected via finger photoplethysmography (NOVA Finapres, Finapres Inc., Enschede, Netherlands) on the dominant arm at heart level. Resting blood pressure was determined via automated sphygmomanometer (Omrom Healthcare, Lake Forest, IL) on the right arm at heart level in the supine position.

Pulmonary gases were recorded utilizing a breath‐by‐breath gas analysis system (CEW Inc., Gemini End‐Tidal O_2_ and CO_2_ Analyzer). Before each testing session, gas exchange equipment was calibrated using known nitrogen and carbon dioxide (CO_2_) gas concentrations.

#### 2.4.1 Cerebral hemodynamic measures

MCAv was continuously recorded via transcranial Doppler ultrasonography using bilateral 2 MHz pulsed‐wave robotic probes affixed to the temporal window by an adjustable headpiece with ultrasound gel (NeuroVision, Multigon Industries, Flint, TX). Ideal vessel insonation angle, depth (between 45 and 65 mm), and orientation were acquired following published guidelines (Aaslid et al., 1982; Willie et al., 2011). Left and right MCAv were recorded and then averaged when possible; otherwise, the best signal from either the left or right side was used for analysis. Prefrontal cortex oxygenation (TSI) was continuously collected using a portable near‐infrared spectroscopy device (50 Hz sampling rate) placed on the right forehead (Portalite, Artinis Medical Systems, Elst, Netherlands). By doing so, we were able to assess any oxygenation changes to regions of the brain fed by the MCA.

#### 2.4.2 Lower body negative pressure

Lower‐body negative pressure (Technavance, Austin, TX, USA) was used to invoke a light (−20 mmHg) hypovolemic response in participants. Participants were sealed into the chamber at the iliac crest while lying in a supine position with arms resting at heart level. To ensure a proper seal prior to data collection, participants were briefly subjected to LBNP and instructed to indicate if a leak was present around their waist. If a quality seal was created, LBNP was turned off and participants were allowed to return to resting conditions for at least 15 min. This stimulus was chosen to stress hemodynamic control while limiting the effects of systemic blood pressure on MCAv responses—as changes in MAP and Q can greatly influence cerebral flow (Claassen et al., 2021). Blood pressure during LBNP of −15 to −20 mmHg has been shown to be easily compensable by the body (Rickards, 2015). By doing so, we hoped to isolate the local vascular effects of CYP450 inhibition without confounding systemic changes in pressure and volume. Moreover, MCA diameter has been shown to remain steady during −20 mmHg of pressure when assessed through MRI, allowing for more accurate and valid measurement of velocity by transcranial doppler (Serrador et al., 2000).

### 2.5 Data processing

All data, except for prefrontal cortex saturation, was collected and integrated using Powerlab, then stored in the acquisition software LabChart (ADInstruments, Sydney, Australia) at a sampling rate of 1 kHz. Prefrontal cortex saturation was collected utilizing manufacturer software on a separate computer at a sampling rate of 10 Hz. MCAv and central cardiovascular variables were averaged on a beat‐by‐beat basis and then binned into 3‐s intervals using Excel (Microsoft Corporation, Redmond, WA, USA). CVCi was calculated as the quotient of MCAv and MAP. To account for body size, stroke volume index (SVi) and cardiac output index (Qi) were calculated by dividing SV or Q by estimated body surface calculated using previously validated methods (Ashley et al., 2020; Yu et al., 2010). Change (Δ) values were calculated by subtracting absolute MCAv, CVCi, and TSI values during steady‐state LBNP from steady‐state resting values.

#### 2.5.1 Transfer function analysis

Beat‐to‐beat MCAv and MAP data were additionally extracted for transfer function analysis (TFA) to calculate gain, phase, and coherence between the two signals using commercially available software (Ensemble R, Elucimed, Wellington, New Zealand) and by following previously published guidelines (Panerai et al., 2023). Data were visually inspected, with spline interpolation to remove physiological calibration signals from finger photoplethysmography waveforms, as well as to resample the data at 4 Hz. Spectral and time‐frequency analysis was based on the Welch algorithm, with each 5‐min segment of data being divided into five 100 s windows with 50% overlap, which was then passed through a Hanning window prior to fast Fourier transform. The cross‐spectrum between MAP and MCAv was determined and then divided by the MAP auto‐spectrum to derive MAP‐MCAv coherence, phase (radians), and gain (cm•s^−1^•mmHg^−1^) within the low frequency (0.07–0.20 Hz) range. Only participants who met the calculated critical coherence threshold of 0.34 were included in the final TFA analysis to ensure reliability of the derived parameters (Panerai et al., 2023).

#### 2.5.2 Critical closing pressure, resistance area product, and subcomponent analysis of autoregulation

Critical closing pressure (CrCP), resistance area product (RAP), and subcomponent analysis of autoregulation were performed on the best MCAv signal for each subject and were analyzed as previously described (Beishon et al., 2018; Panerai et al., 2025). In short, raw MCAv and MAP data were visually inspected for large, nonphysiological spikes and for finger photoplethysmography calibration signals, which were removed by linear interpolation. Next, the MCAv signal was passed through a median filter to remove any smaller spikes within the data. All data were then processed through a zero phase, low‐pass Butterworth filter at a cutoff frequency of 20 Hz. ECG R‐R intervals were utilized to denote each cardiac cycle. Over the course of each cardiac cycle, estimates of CrCP and RAP were calculated using the 2‐point mean method (2Pm), then averaged to produce a single value for each subcomponent per beat (Panerai et al., 2011). This method of calculation is preferred due to its low production of negative CrCP values and has been recommended for both dynamic and static applications (Panerai et al., 2011). To develop a uniform, equidistant time base, all beat‐to‐beat averages underwent a spline interpolation and were then resampled at 5 Hz.

Previous research has detailed the methods to calculate MCAv responses in terms of its relative subcomponents (Beishon et al., 2018; Panerai et al., 2025). Resting MCAv (V_0_) can be derived in terms of MAP (MAP_0_), CrCP (C_0_), and RAP (R_0_) at a given timepoint using the following equation (Beishon et al., 2018; Panerai, 2003):

(1) $$V_{0}=\frac{{\mathrm{MAP}}_{0}-C_{0}}{R_{0}}$$

During stress, the change in blood velocity (Δ*V*) can be represented as the sum of all changes in each subcomponent in addition to the associated resting values (Beishon et al., 2018; Panerai, 2003):

(2) $$\begin{array}{c} \left(V_{0}+∆V\right)=\frac{\left({\mathrm{MAP}}_{0}+∆\mathrm{MAP}\right)-\left(C_{0}+∆C\right)}{\left(R_{0}+∆R\right)} \end{array}$$

Thus, the previous equation can be transformed utilizing a first‐order Taylor expansion to produce the estimated percentage contributions of a given change in MCAv (Δ*v* = Δ*V*/*V*_0_) by each of its individual subcomponents *V*_CrCP_, *V*_RAP_, and *V*_MAP_—as explained by the equation (Beishon et al., 2018):

(3) $$\begin{array}{c} ∆v=V_{\text{CrCP}}+V_{\mathrm{RAP}}+V_{\mathrm{MAP}} \end{array}$$

where if the Δ*R* < *R*_0_, each percentage contribution of each subcomponent can be calculated as (Beishon et al., 2018):

(4) $$\begin{array}{c} V_{\text{CrCP}}=-\frac{∆\mathrm{MAP}}{V_{0}R_{0}} \end{array}$$

(5) $$\begin{array}{c} V_{\mathrm{RAP}}=-\frac{∆R}{R_{0}} \end{array}$$

(6) $$\begin{array}{c} V_{\mathrm{Map}}=\frac{∆\mathrm{MAP}}{V_{0}R_{0}} \end{array}$$

Importantly, due to the negative signs within Equations 4 and 5, a rise in *V*_CrCP_ and *V*_RAP_ represents a decrease in the contribution of each respective subcomponent. Similarly, a fall in *V*_CrCP_ and *V*_RAP_ represents an increase in the contribution of each respective subcomponent (Beishon et al., 2018). Data were normalized to a 20 s baseline average of CrCP (*C*_0_), RAP (*R*_0_), and MAP (MAP_0_) prior to LBNP activation.

Area under the curve was calculated for each subcomponent's contribution (AUC_CrCP_, AUC_RAP_, and AUC_MAP_) to a change in velocity across 5 min of LBNP utilizing the trapezoidal method.

### 2.6 Statistical analyses

Due to lack of human data studying CYP450 in the brain, literature utilizing indomethacin (Smirl et al., 2015)—an inhibitor of prostaglandin production—was used as a surrogate to estimate a required sample size needed to reach a power of 0.85 (Gpower 3, Faul et al., 2007). To do this, we estimated the effect of indomethacin on MCAv using group variance between indomethacin and control. This literature was chosen due to its similar instrumentation and use of a drug that influences endothelial pathways that complement nitric oxide's role in the brain's vasculature, like CYP450. By doing so, an estimated sample size of 14 was determined.

#### 2.6.1 Resting and steady state statistical analyses

All steady state variables were compared with averages of the last 30 s of each stage, except for CrCP and RAP, which were averaged over the last 20 s of each stage as previously described (Beishon et al., 2018). Normality of data was determined using Shapiro–Wilk tests. All group effects and interactions within central and cerebrovascular data were calculated using a two‐way repeated measure ANOVA (within subject effects: time and treatment), followed by a Bonferroni‐adjusted post hoc test, as required. Effect size for group effects and interaction effects was calculated using partial eta squared ($\eta_{p}^{2}$). Each Bonferroni post hoc's effect size was calculated using Cohen's *d*. Moreso, changes from baseline (Δ values) were compared using paired *t*‐tests, or Mann–Whitney *U* tests if data was non‐normally distributed. Effect size for *t*‐test results were calculated as Cohen's *d* or effect size *r*. Variance of Δ values were calculated as the standard deviation squared.

To ensure that there was no effect of end tidal CO_2_ on the model, a mixed‐effect, random intercept model was performed on cerebrovascular variables (fixed effects: time, treatment, time × treatment, and partial pressure of end tidal carbon dioxide (PETCO_2_), random effects: subject).

#### 2.6.2 Dynamic cerebral autoregulation statistical analyses

All group effects and interactions in TFA (gain, phase, and coherence) were calculated using a two‐way repeated‐measures ANOVA (within subject effects: time and treatment), followed by Bonferroni post hoc tests as needed. Effect size for group effects and interaction effects was calculated using partial eta squared ($\eta_{p}^{2}$). Differences in steady state subcomponent (V_CrCP_, V_RAP_, and V_MAP_) activity during the last 20 s of LBNP and the area under the curve for each subcomponent response (AUC_CrCP_, AUC_RAP_, AUC_MAP_) were compared via two‐tailed, paired Student's *t*‐test. If data was not normally distributed, data was compared using a Wilcoxon signed‐rank test. Cohen's *d* or effect size *r* was utilized for effect size of normal and non‐normal data, respectively.

#### 2.6.3 Sex differences statistical analyses

All group and interaction effects within steady state and subcomponent variables were determined using a mixed‐methods three‐way repeated‐measures ANOVA (within subject effects: time and treatment, between subject effects: sex), followed by a Bonferroni‐adjusted post hoc test as required. Effect size for group effects and interaction effects was calculated using partial eta squared ($\eta_{p}^{2}$). Each Bonferroni post hoc's effect size was calculated using Cohen's *d*.

Due to having an inadequate number of subject data that met the coherence threshold, TFA variables were not analyzed for sex differences.

All statistical analyses were analyzed in R Studio (v2024.04.2, Posit Software 2024). For non‐normally distributed data, effect size was calculated as $r=Z/\left(\sqrt{n_{1}+\;n_{2}}\right)$. Small effect sizes for *r, d*, and $\eta_{p}^{2}$ were 0.10, 0.20, and 0.01, respectively. Moderate effect sizes for *r, d*, and $\eta_{p}^{2}$ were 0.30, 0.50, and 0.06, respectively. Large effect sizes for *r, d*, and $\eta_{p}^{2}$ were 0.50, 0.80, and 0.14, respectively. Data are represented as mean ± SD. Alpha was set at ≤0.05.

## 3 RESULTS

### 3.1 Subject characteristics

Participant anthropometric data can be viewed in Table 1. There were no differences between the sexes with respect to age, body mass index (BMI), waist circumference, hip circumference, or diastolic blood pressure (DBP) (*p* > 0.05). Males demonstrated larger height (*p* = < 0.001), weight (*p* = 0.013), and systolic blood pressure (SBP) (*p* = 0.002) than females.

**TABLE 1:** Participant characteristics.

	Whole sample	Males	Females	*p* Value
	(*n* = 23)	(*n* = 11)	(*n* = 12)	
Age (years)	21.6 (2.9)	22.3 (2.5)	21.0 (3.1)	0.292
Height (cm)	173.6 (11.5)	181.9 (9.9)	166.0 (6.6)^a^	<0.001
Weight (kg)	72.7 (13.7)	80.0 (14.7)	65.9 (8.6)^a^	0.013
BMI (kg·m^−2^)	24.4 (3.1)	24.5 (3.2)	24.2 (3.1)	0.846
Waist Circum. (cm)	81.6 (8.7)	82.4 (8)	80.8 (9.6)	0.678
Hip Circum. (cm)	100.9 (8.8)	98.9 (10.6)	102.7 (6.7)	0.318
Systolic BP (mmHg)	115.3 (9.9)	121.2 (6.6)	110.0 (8.9)^a^	0.002
Diastolic BP (mmHg)	70.1 (5.9)	68.4 (6.2)	71.5 (5.4)	0.207
Contraceptives	Oral	*n* = 4	Patch	n = 1
	IUD	n = 1	None	*n* = 6

*Note*: Values are mean ± SD.

Abbreviations: BMI, body mass index; BP, blood pressure; Circum., circumference; IUD, intrauterine device.

^a^ Denotes significant difference between sexes utilizing a Student's *t*‐test, *p* < 0.05.

### 3.2 Resting and steady state central cardiopulmonary responses to LBNP

There were no significant differences between PLC and FLZ resting central cardiopulmonary measures following FLZ administration (Table 2).

**TABLE 2:** Repeated measures ANOVA results.

	PLC		FLZ		RM ANOVA					
	Baseline	LBNP	Baseline	LBNP	Time		Treatment		Time × Treatment	
					*p* Value	$\eta_{p}^{2}$	*p* Value	$\eta_{p}^{2}$	*p* Value	$\eta_{p}^{2}$
*n* = 23										
MCAv (cm·s^−1^)	74.6 (11.9)	71.5 (12.0)	75.2 (11.3)	70.6 (11.2)^b^	<0.001^a^	0.528	0.936	<0.001	0.451	0.026
CVCi (cm·s^−1^·mmHg^−1^)	0.75 (0.13)	0.72 (0.14)	0.76 (0.12)	0.71 (0.11)^b^	<0.001^a^	0.612	0.897	<0.001	0.215	0.069
TSI (%)	68.4 (6.0)	68.4 (5.6)	68.6 (7.5)	68.2 (7.1)	0.338	0.042	0.983	<0.001	0.286	0.052
*n* = 21										
CrCP (mmHg)	29.7 (9.3)	25.1 (11.3)^b^	26.5 (10.0)	24.1 (8.4)	<0.001^a^	0.459	0.438	0.030	0.192	0.083
RAP (mmHg·s·cm^−1^)	0.96 (0.29)	1.08 (0.32)^b^	0.98 (0.24)	1.07 (0.23)^b^	<0.001^a^	0.732	0.831	0.002	0.293	0.055
*n* = 23										
MAP (mmHg)	100.2 (9.2)	100.0 (9.0)	99.4 (8.2)	100.5 (8.4)	0.560	0.016	0.962	<0.001	0.348	0.040
Q (L· min^−1^)	5.4 (1.1)	5.1 (1.2)	5.4 (1.6)	5.4 (1.5)	0.070	0.141	0.401	0.032	0.550	0.017
HR (bpm)	61.1 (6.6)	67.2 (9.3)^b^	60.1 (5.1)	68.8 (8.9)^b^	<0.001^a^	0.728	0.805	0.003	0.218	0.068
SV (mL)	85.7 (18.0)	74.2 (19.6)^b^	87.0 (21.4)	75.2 (21.9)^b^	<0.001^a^	0.682	0.738	0.005	0.894	<0.001
Qi (L· min^−1^· m^2^)	2.90 (0.53)	2.75 (0.50)	2.96 (0.71)	2.88 (0.70)	0.075	0.137	0.474	0.024	0.423	0.029
SVi (mL· beat · m^2^)	45.83 (7.2)	39.7 (8.5)^b^	46.4 (8.7)	40.2 (10.4)^b^	<0.001^a^	0.684	0.765	0.004	0.961	<0.001
TPR (mmHg·min·L^−1^)	19.3 (4.4)	20.4 (5.2)	19.4 (5.6)	20.3 (6.6)	0.045^a^	0.170	0.971	<0.001	0.713	<0.001
PETCO_2_ (mmHg)	40.9 (5.7)	39.5 (6.3)^b^	42.1 (4.1)	40.9 (5.2)	<0.001^a^	0.396	0.416	0.030	0.792	0.003

*Note*: Values are mean ± SD.

Abbreviations: CrCP, critical closing pressure; CVCi, cerebrovascular conductance index; FLZ, fluconazole condition; HR, heart rate; LBNP, lower body negative pressure; MAP, mean arterial pressure; MCAv, middle cerebral artery velocity; PETCO_2_, partial pressure of end tidal carbon dioxide; PLC, placebo condition; Q, cardiac output; Qi, cardiac output index; RAP, resistance area product; SV, stroke volume; SVi, stroke volume index; TPR, total peripheral resistance; TSI, prefrontal cortex tissue saturation index; $\eta_{p}^{2}$, partial eta squared.

^a^ Denotes significant effect utilizing a 2 × 2 repeated measures ANOVA; post hoc testing was performed via Bonferroni tests.

^b^ Denotes difference between baseline and LBNP within treatment, *p* < 0.05.

MAP and Q were not changed following LBNP activation for both PLC and FLZ (Table 2). However, HR and TPR increased from baseline during steady state LBNP activation for both PLC and FLZ but observed no effect of treatment or time × treatment interaction (Table 2). Although TPR showed a main effect of time, post hoc testing could not detect where this effect lies when comparing the means. PETCO_2_ demonstrated a significant decrease from baseline following LBNP activation during PLC, but not in FLZ, and showed no effect of treatment or time × treatment interaction (PLC_BASE_ 40.9 ± 5.69 mmHg vs. PLC_LBNP_ 39.5 ± 6.28 mmHg, *p =* 0.004, *d* = 0.826, Table 2). LBNP resulted in diminished steady state SV from baseline in both PLC and FLZ but did not show a treatment or time × treatment effect (PLC_BASE_ 85.7 ± 18.0 mL vs. PLC_LBNP_ 74.2 ± 19.6 mL, *p* = <0.001, *d* = 1.57; FLZ_BASE_ 87.0 ± 21.4 mL vs. FLZ_LBNP_ 75.2 ± 21.9 mL, *p* = 0.002, *d* = 0.906, Table 2).

### 3.3 Resting and steady state cerebral hemodynamic responses to LBNP

There were no significant differences in resting cerebral hemodynamic measures between PLC and FLZ administration (Table 2).

**FIGURE 1:**
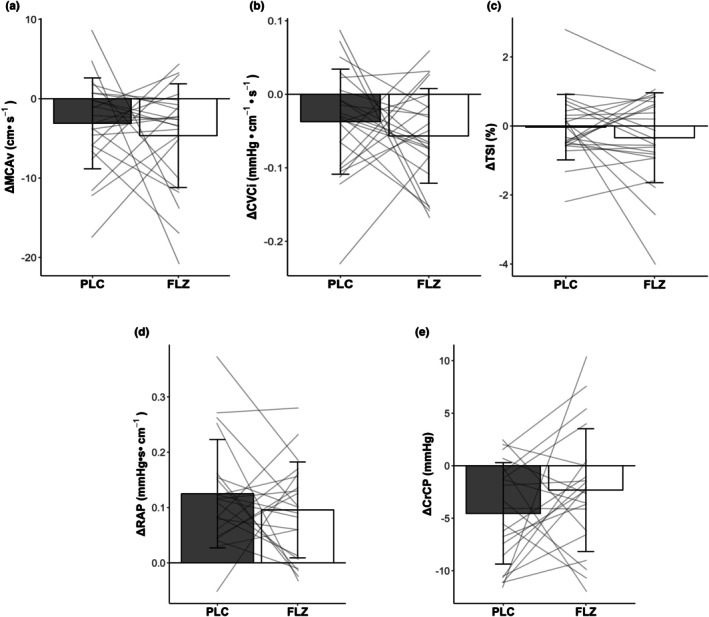
Values are mean ± SD. Grouped and individual (a–c, *n* = 23; d, e, *n* = 21) change values in (a) middle cerebral artery velocity, (b) cerebrovascular conductance index (MCAv/MAP), (c) prefrontal cortex oxygen saturation, (d) resistance area product, and (e) critical closing pressure from rest (gray bars) to steady state −20 mmHg lower‐body negative pressure (white bars) between sexes (female: Dot) and treatment. Bars represent mean. All analysis was performed using a paired *t*‐tests or Mann–Whitney U test. CrCP, critical closing pressure; CVCi, cerebrovascular conductance index; FLZ, fluconazole visit; MCAv, middle cerebral artery velocity; PLC, placebo visit; RAP, resistance area product; TSI, prefrontal cortex oxygen saturation.

LBNP elicited a decrease in MCAv from baseline only within FLZ but did not result in a treatment or time × treatment effect (PLC Δ −3.10 ± 5.73 cm•s^−1^ vs. FLZ Δ −4.66 ± 6.54 cm•s^−1^, Table 2). Additionally, LBNP decreased CVCi from baseline only within FLZ and did not result in a treatment or time × treatment effect (PLC Δ −0.04 ± 0.07 cm•s^−1^•mmHg^−1^ vs. FLZ Δ −0.06 ± 0.06 cm•s^−1^•mmHg^−1^, Table 2). LBNP did not affect pre‐frontal cortex TSI in both PLC and FLZ, and did not result in a time, treatment, or time*treatment interaction (PLC Δ −0.03 ± 0.95% vs. FLZ Δ −0.34.2 ± 1.3%, Table 2). Further, ΔMCAv, ΔCVCi, and ΔTSI were not different from each other (*p* = 0.571, *r* = 0.03; *p* = 0.405, *d* = 0.18; *p* = 0.556, *r* = 0.02, respectively; Figure 1a–c). Although both treatments demonstrated the same degree of variability, variation in responses to LBNP were large (ΔMCAv PLC 32.83 cm^2^•s^−2^ vs. FLZ 42.77 cm^2^•s^−2^; ΔCVCi PLC 0.005 cm^2^•s^−2^•mmHg^−2^ vs. FLZ 0.004 cm^2^•s^−2^•mmHg^−2^; ΔTSI PLC 0.90%^2^ vs. FLZ 1.69%^2^).

Due to interference from extended blood pressure physiological calibration signals, two participants were excluded from absolute CrCP and RAP calculations (total: 9 males, 12 females). During LBNP absolute CrCP was reduced from baseline only within PLC but did not show an effect of treatment or a time × treatment interaction (PLC Δ −4.54 ± 4.83 mmHg vs. FLZ Δ −2.32 ± 5.84 mmHg, Table 2). RAP was reduced during LBNP in PLC and FLZ, demonstrating an effect of time but not an effect of treatment or a time × treatment interaction (PLC Δ 0.13 ± 0.29 mmHg•s•cm^−1^ vs. FLZ Δ 0.10 ± 0.09 mmHg•s•cm^−1^, Table 2). ΔCrCP and ΔRAP did not differ (*p* = 0.267, *d* = 0.17; *p* = 0.405, *d* = 0.06, respectively; Figure 1d,e). Although both treatments demonstrated the same degree of variability, variation in responses to LBNP were large (ΔCrCP PLC 23.33 mmHg^2^ vs. FLZ 34.11 mmHg^2^; ΔRAP PLC 0.01 mmHg^2^•s^2^•cm^−2^ vs. FLZ 0.008 mmHg^2^•s^2^•cm^−2^).

Analysis accounting for end tidal CO_2_ revealed similar results, where there was a significant effect of time on MCAv (*β* = −4.55, SE = 1.68, *t* = −2.71, *p* = 0.009, Table 3), but no effect of treatment, PETCO_2_, or a time × treatment interaction. Moreso, linear mixed effect modeling also demonstrated a significant effect of time on CVCi (*β* = −0.06, SE = 0.02, *t* = −2.70, *p* = 0.009, Table 3), but no effect of treatment, PETCO_2_, or a time × treatment interaction. Linear mix‐effects modeling did not show any effects of time, treatment, PETCO_2_, or time × treatment interactions for TSI (Table 3). Unsurprisingly, only PETCO_2_ displayed a main effect on CrCP (*β* = 0.45, SE = 0.19, *t* = 2.42, *p* = 0.018, Table 3). Linear mixed modeling demonstrated a main effect of time for RAP (*β* = 0.09, SE = 0.04, *t* = 2.27, *p* = 0.027, Table 3).

**TABLE 3:** Mixed effect linear model—Cerebrovascular variables.

	Fixed effects	*β*	SE	*t*	*p* Value
MCAv (cm•s^−1^)	(intercept)	71.45	6.85	10.43	<0.001^a^
	Time	−4.55	1.68	−2.71	0.009^a^
	Treatment	−0.54	1.68	−0.32	0.747
	PETCO_2_	0.09	0.15	0.59	0.555
	Time × Treatment	1.57	2.36	0.67	0.507
CVCi (cm•s^−1^•mmHg^−1^)	(intercept)	0.79	0.08	9.52	<0.001^a^
	Time	−0.56	0.02	−2.70	0.009^a^
	Treatment	−0.01	0.02	−0.54	0.593
	PETCO_2_	−0.001	0.002	−0.38	0.703
	Time × Treatment	0.03	0.03	0.90	0.374
TSI (%)	(intercept)	74.58	4.03	18.52	<0.001^a^
	Time	−0.52	0.99	−0.52	0.604
	Treatment	−0.30	1.00	−0.30	0.776
	PETCO_2_	−0.14	0.09	−1.59	0.116
	Time × Treatment	0.28	1.40	0.20	0.840
CrCP (mmHg)	(intercept)	7.31	8.18	0.89	0.374
	Time	−1.74	2.30	−0.76	0.450
	Treatment	3.98	2.31	1.73	0.090
	PETCO_2_	0.45	0.19	2.42	0.018
	Time × Treatment	−2.27	3.23	−0.70	0.485
RAP (mmHg•s•cm^−1^)	(intercept)	1.02	0.17	6.15	<0.001
	Time	0.09	0.04	2.27	0.027
	Treatment	−0.03	0.04	−0.63	0.533
	PETCO_2_	−0.001	0.003	−0.26	0.794
	Time × Treatment	0.03	0.06	0.50	0.617

*Note*: Values are mean ± SD.

Abbreviations: CrCP, critical closing pressure; CVCi, cerebrovascular conductance index; MCAv, middle cerebral artery velocity; PETCO_2_, partial pressure of end tidal carbon dioxide; RAP, resistance area product; TSI, prefrontal cortex tissue saturation.

^a^ Denotes significant effect utilizing a mixed effect linear model, *p* ≤ 0.05.

### 3.4 Dynamic autoregulatory responses to LBNP

**FIGURE 2:**
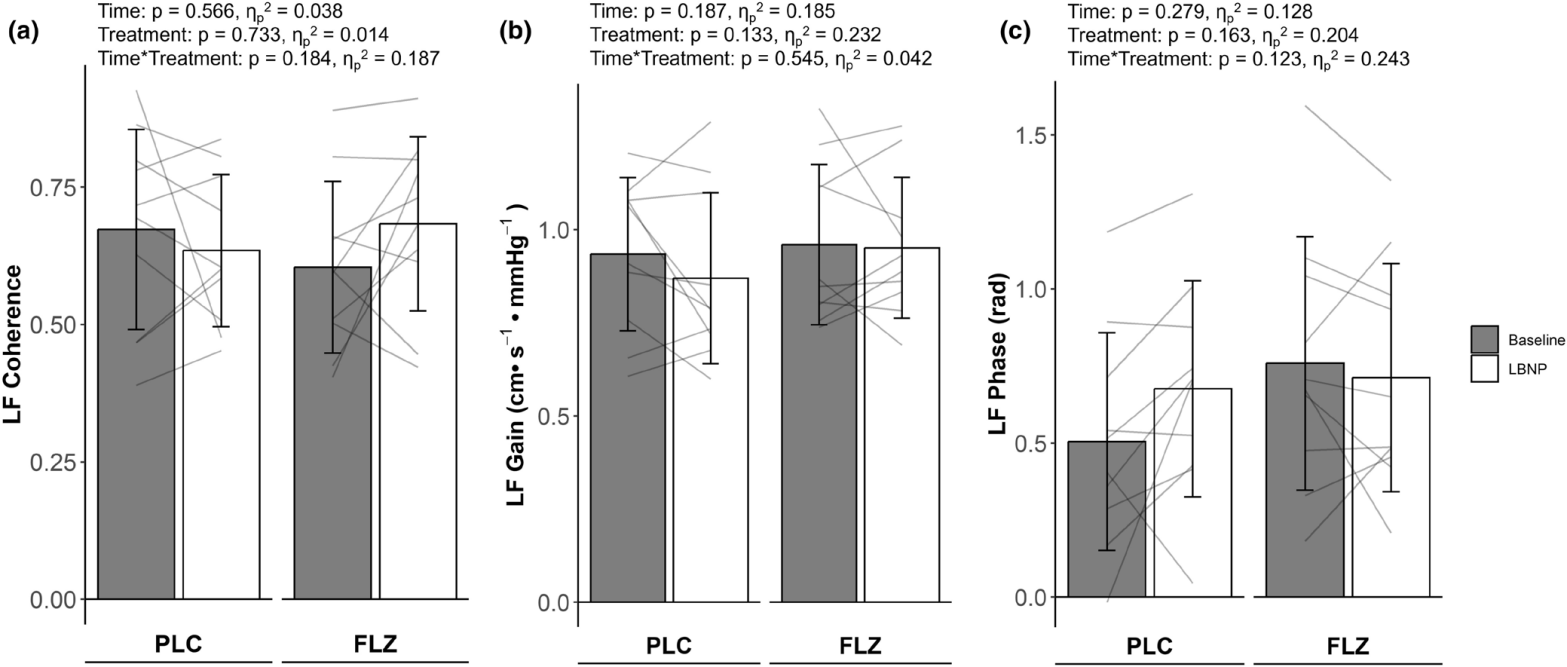
Values are mean ± SD. Transfer function analysis during rest (gray bars, *n* = 10) and −20 mmHg lower‐body negative pressure (white bars, *n* = 10) between PLC and FLZ. Low frequency coherence (a), gain (b), and phase (c) are represented as means (bars) and individual responses (lines). All analysis was performed using repeated measures ANOVA. FLZ, fluconazole visit; LF, low frequency band; PLC, placebo visit.

Thirteen participants were excluded from transfer function analysis due to low coherence values (coherence value <0.34). Because of this, only 10 participants (total: 3 males, 7 females) were analyzed for gain and phase comparison. Low frequency coherence between participants reported no effect of time, treatment, or interaction (PLC_BASE_ 0.67 ± 0.18 vs. PLC_LBNP_ 0.64 ± 0.14; FLZ_BASE_ 0.60 ± 0.16 vs. FLZ_LBNP_ 0.68 ± 0.16, Figure 2a). Within the low frequency band, gain showed no effect of time, treatment, or interaction between participants (PLC_BASE_ 0.93 ± 0.21 cm•s^−1^ • mmHg^−1^ vs. PLC_LBNP_ 0.87 ± 0.23 cm•s^−1^ • mmHg^−1^; FLZ_BASE_ 0.96 ± 0.22 cm•s^−1^ • mmHg^−1^ vs. FLZ_LBNP_ 0.95 ± 0.19 cm•s^−1^ • mmHg^−1^, Figure 2b). Moreover, low frequency phase showed no effect of time, treatment, or interaction between participants (PLC_BASE_ 0.51 ± 0.35 radians vs. PLC_LBNP_ 0.68 ± 0.35 radians; FLZ_BASE_ 0.76 ± 0.41 radians vs. FLZ_LBNP_ 0.71 ± 0.37 radians, Figure 2c).

**FIGURE 3:**
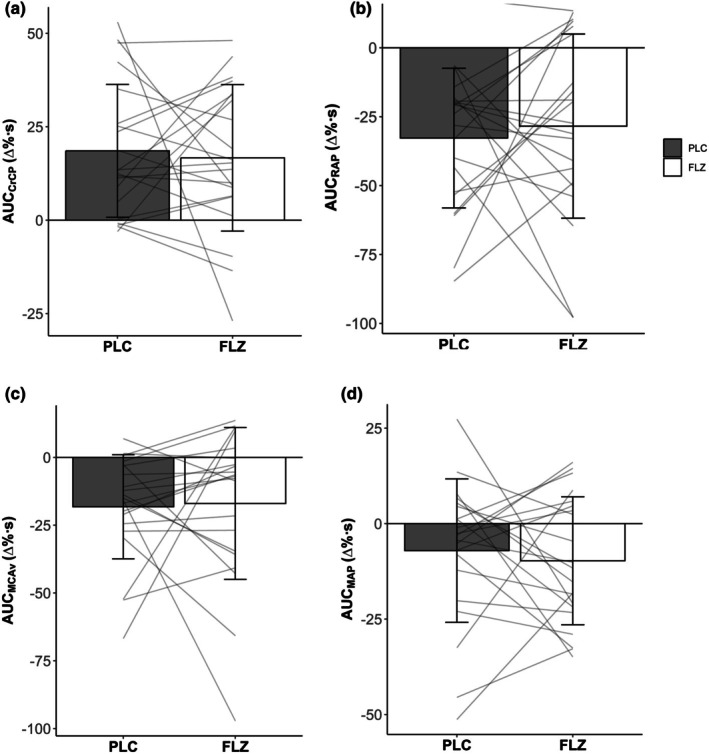
Values are mean ± SD. Grouped and individual (*n* = 21) area under the curve responses during 5 min of −20 mmHg LBNP between PLC and FLZ. (a) area under the curve for percent contribution in CrCP, (b) area under the curve for the percent contribution of RAP, (c) area under the curve for the percent change in MCAv, (d) area under the curve for the percent contribution of MAP. All analysis was performed using a mixed model repeated measure ANOVA. AUC_CrCP_, total contribution to velocity by critical closing pressure, AUC_MAP_, total contribution to velocity by mean arterial pressure; AUC_MCAv_, total change in middle cerebral artery velocity; AUC_RAP_, total contribution to velocity by resistance area product; FLZ, fluconazole visit; PLC, placebo visit.

Two participants were excluded from subcomponent analysis due to poor data quality due to extended blood pressure calibration signals that overlapped between stages. Because of this, only 21 participants (total: 9 males, 12 females) were included for analysis. Subcomponent contributions to the maintenance of flow during steady state LBNP were not different between treatments (PLC V_MAP_ 0.61 ± 7.81Δ% vs. FLZ V_MAP_ 0.60 ± 8.13Δ%, *p =* 0.200, *d* = <0.001; PLC V_CrCP_ 6.60 ± 6.98Δ% vs. FLZ V_CrCP_ 3.50 ± 7.96Δ%, *p =* 0.998, *d* = 0.289; PLC V_RAP_ −32.7 ± 25.4Δ% vs. FLZ V_RAP_ −28.4 ± 33.4Δ%, *p =* 0.419, *d* = 0.180). Although LBNP resulted in a total decreased contribution of CrCP and an increased contribution in RAP in the maintenance of blood velocity across lower body negative pressure, there were no differences between the total effect of treatments (PLC AUC_CrCP_ 18.6 ± 17.8 Δ%•s vs. FLZ AUC_CrCP_ 16.7 ± 19.6 Δ%•s, *p* = 0.742, *d* = 0.29; PLC AUC_RAP_ −32.7 ± 25.4 Δ%•s vs. FLZ AUC_RAP_ −28.4 ± 33.4 Δ%•s, *p* = 0.550, *r* = 0.02, Figure 3a,b). Similarly, there was no statistical difference in the contribution of MAP on MCAv response during 5 min of −20 mmHg LBNP (PLC AUC_MAP_ −7.05 ± 18.8 Δ%•s vs. FLZ AUC_MAP_ −9.73 ± 16.8 Δ%•s, *p* = 0.595, *d* = 0.001, Figure 3d).

### 3.5 Sex differences

No central cardiac variables resulted in any time × sex or time × treatment × sex interactions. However, Q (Males 5.90 ± 1.61 L•min^−1^•m^2^ vs. Females 4.88 ± 0.92 61 L•min^−1^•m^2^, Sex: *p* = 0.050, $\eta_{p}^{2}$ = 0.172), SV (Males 91.8 ± 20.8 mL•beat^−1^ vs. Females 70.2 ± 14.7 mL•beat^−1^, Sex: *p* = 0.002, $\eta_{p}^{2}$ = 0.375), PETCO_2_ (Males 42.5 ± 5.98 mmHg vs. Females 39.4 ± 4.29 mmHg, Sex: *p* = 0.047, $\eta_{p}^{2}$ = 0.175), and HR (Males 61.6 ± 7.63 bps vs. Females 66.8 ± 8.42 bps, Sex: *p* = 0.038, $\eta_{p}^{2}$ = 0.190) demonstrated a main effect of sex, where Q, SV, PETCO_2_ were higher in males, while HR was higher in females. Normalizing SV and Q to body surface area removed the effect of sex.

**FIGURE 4:**
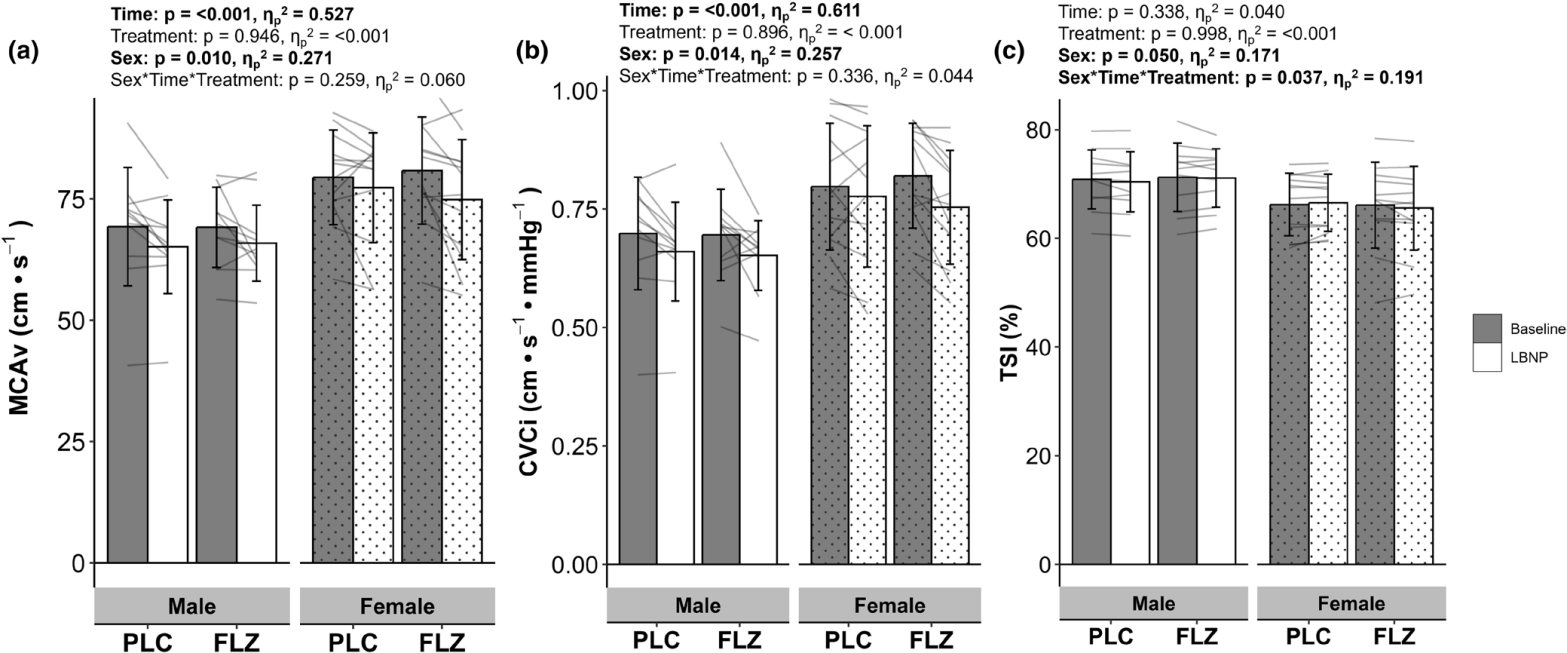
Values are mean ± SD. Grouped and individual (*n* = 23) absolute values in (a) middle cerebral artery velocity, (b) cerebrovascular conductance index (MCAv/MAP), and (c) prefrontal cortex oxygen saturation from resting steady state (gray bars) to steady state −20 mmHg lower‐body negative pressure (last 30 s of LBNP, white bars) between sexes (female: Dot) and treatment. Bars represent mean. All analysis was performed using a mixed model repeated measure ANOVA. CVCi = cerebrovascular conductance index, FLZ, fluconazole visit; MCAv, middle cerebral artery velocity; PLC, placebo visit; TSI, prefrontal cortex oxygen saturation.

**FIGURE 5:**
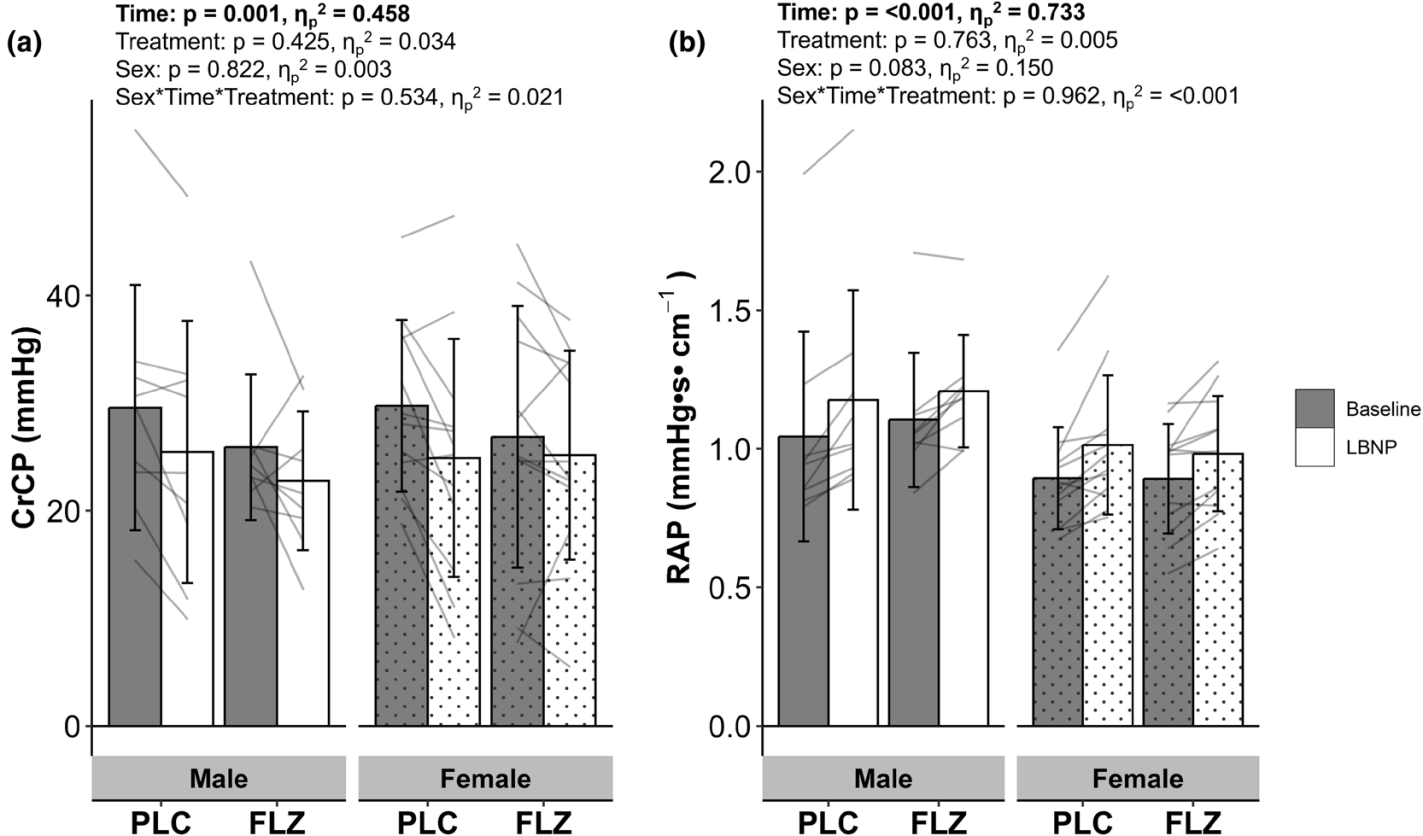
Values are mean ± SD. Grouped and individual (*n* = 21) absolute values in (a) critical closing pressure, (b) resistance area product from resting steady state (gray bars) to steady state −20 mmHg lower‐body negative pressure (last 20 s of LBNP, white bars) between sexes (female: Dot) and treatment. Bars represent mean. All analysis was performed using a mixed model repeated measure ANOVA. CrCP, critical closing pressure; FLZ, fluconazole visit; PLC, placebo visit; RAP, resistance area product.

There was a significant effect of sex on MCAv (Males 67.37 ± 9.49 cm•s^−1^ vs. Females 78.12 ± 11.03 cm•s^−1^), however, there was no effect of sex × treatment, sex × time, or sex × treatment × time (Figure 4a). Similarly, there was a main effect of sex on CVCi (Males 0.68 ± 0.10 cm•s^−1^•mmHg^−1^ vs. Females 0.79 ± 0.13 cm•s^−1^•mmHg^−1^), with no sex × treatment, sex × time, or sex × treatment × time interaction (Figure 4b). TSI was influenced by sex and demonstrated a sex × treatment × time interaction, however post hoc testing was unable to identify where this interaction occurs (Figure 4c). There were no effects of sex, sex × treatment, or sex × time × treatment interactions for the absolute values of CrCP and RAP (Figure 5a,b).

**FIGURE 6:**
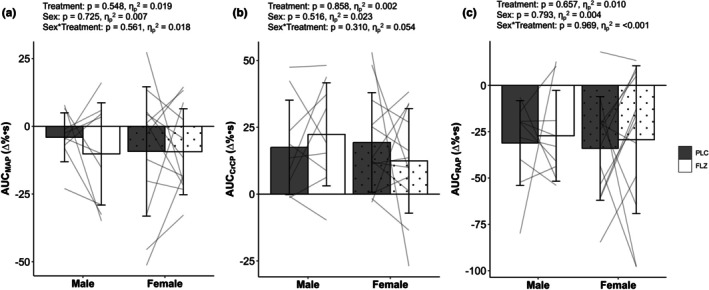
Values are mean ± SD. Grouped and individual (*n* = 21) area under the curve responses during 5 min of −20 mmHg LBNP between sexes (female: Dot) and treatment (PLC: Gray bars, FLZ: White bars). (a) area under the curve for percent contribution in MAP, (b) area under the curve for the percent contribution of CrCP, (c) area under the curve for the percent change in RAP. All analysis was performed using a mixed model repeated measure ANOVA. AUC_CrCP_, total contribution to velocity by critical closing pressure, AUC_RAP_, total contribution to velocity by resistance area product; AUC_MAP_, total contribution to velocity by mean arterial pressure; FLZ, fluconazole visit; PLC, placebo visit.

**FIGURE 7:**
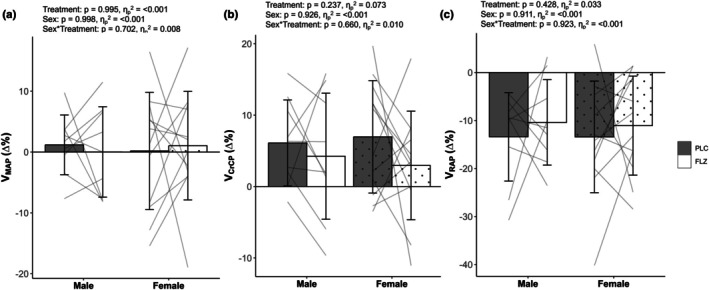
Values are mean ± SD. Grouped and individual (*n* = 21) relative contributions of (a) MAP, (b) CrCP, and (c) RAP to steady state MCAv responses during the last 20 s of LBNP between sexes (female: Dot) and treatment (PLC: Gray bar, FLZ: White bar). FLZ, fluconazole visit; PLC, placebo visit; V_CrCP_, percent contribution to velocity by critical closing pressure; V_RAP_, percent contribution to velocity by resistance area product; V_MAP_, percent contribution to velocity by mean arterial pressure.

Due to small sample size and unbalanced groups, sex differences between TFA variables were unable to be analyzed. However, dynamic autoregulation assessed through AUC of autoregulatory subcomponents showed there was no effect of sex, treatment, or sex × treatment interaction for AUC variables during LBNP (Figure 6a–c). There was no effect of treatment, sex, or sex × treatment interaction for V_MAP_, V_RAP_, or V_CrCP_ (Figure 7a–c).

## 4 DISCUSSION

The main findings of this study were (1) CYP450 inhibition did not affect resting cerebral hemodynamics in healthy young participants, (2) although not statistically different between treatments and reporting no interaction effects, only CYP450 inhibition obstructed static cerebrovascular regulation during LBNP in healthy young individuals, (3) CYP450 inhibition did not affect dynamic cerebral autoregulation in healthy young individuals as assessed through transfer function analysis and subcomponent analysis of autoregulation and (4) there was no difference in steady state responses to LBNP between the sexes with regard to static or dynamic cerebral blood flow measures—but blockade of CYP450 did present a significant sex × time × treatment interaction in subject prefrontal cortex saturation. However, where this difference lies within the means was undetectable with post hoc testing.

Therefore, these data allude to altered metabolic control of cerebral hemodynamics during CYP450 inhibition (e.g., FLZ) as assessed through within‐group reductions in MCAv and CVCi, coinciding with a lack of CrCP response during LBNP. Thus, CYP450 appears to potentially contribute to, but is not required for static cerebral hemodynamic regulation in healthy, young adults. To our knowledge, this is the first study to directly study the effect of CYP450 blockade on human cerebrovascular control and therefore provides a foundation for future research regarding CYP450's role in cerebrovascular control during aging and disease.

### 4.1 Resting and steady‐state cerebrovascular responses

Unlike previous animal models (Alkayed et al., 1996), the current study found that resting cerebral hemodynamics did not differ following CYP450 inhibition compared to placebo controls. Morever, steady state hemodynamics were similar between treatments during slight hypovolemic stress (−20 mmHg LBNP). These contradictions likely reflect inherent methodological differences between studies, as well as a lack of adequate power to properly identify the delicate role of CYP450 in the healthy, young cerebrovasculature.

First, the current study performed a systemic dose of fluconazole compared to a localized dose of miconazole to the brain's dura mater—resulting in a less potent and more systemic inhibition of CYP450 in the brain's neuronal, glial, and vascular endothelium cells due to fluconazole's ability to pass the blood‐brain barrier and saturate cerebrospinal fluid (Grant & Clissold, 1990). Second, in Alkayed and colleagues' experiment, they measured microvascular perfusion of the brain using laser‐doppler flowmetry, while we quantified flow utilizing middle cerebral artery velocity through transcranial Doppler ultrasonography. Inherently, this technique allows for the measurement of blood velocities through large conduit vessels and does not directly measure changes in the microvasculature (Tyagi et al., 2000). Still, changes in the downstream microvascular environment often present changes in velocity through larger vessels due to the large influence the microvasculature has on vascular resistance in the brain (Cipolla, 2009). In the current study, Bonferroni testing revealed that MCAv and CVCi were attenuated only within the FLZ treatment—suggesting some alteration in hemodynamics during LBNP following treatment.

In agreement with this, Bonferroni testing in the current study demonstrated that hypovolemic stress did not reduce CrCP in FLZ. PLC, on the other hand, did show lowered CrCP during −20 mmHg LBNP. This likely signifies impaired metabolic control downstream of the MCA in response to hypovolemic stress, as critical closing pressure is considered to reflect metabolically mediated changes in the microvascular environment (Ince et al., 2023). This data agrees with previous in vitro animal models, which demonstrate that CYP450 metabolites predominantly affect smaller caliber vessels rather than larger arteries (Davis et al., 2011; Dietrich et al., 2009; You et al., 1999). Additionally, LBNP resulted in a robust increase in RAP in both treatment groups with a moderate effect size, demonstrating that small arteries and large arterioles, where myogenic activity would be most prevalent, increased activity during −20 mmHg LBNP. Thus, it appears that when quantified using CrCP and RAP, it is likely that there is increased metabolically mediated dilation further down the vascular tree to oppose myogenically mediated constrictions of the larger arteries during bouts of mild hypovolemic stress. Intervention with CYP450 inhibition likely attenuated this metabolically mediated dilation while increased myogenic constriction persisted, thus impairing static regulation of MCAv—of which only FLZ treatment demonstrated a significant hindrance.

### 4.2 Dynamic cerebrovascular responses

Previous work in rats has shown a strong connection between CYP450‐mediated increases in HETE‐20 production, cerebral myogenic response, and rising blood pressures in vitro (Gebremedhin et al., 2000). Gebremedhin et al. (2000) demonstrated that inhibition of HETE‐20 production attenuates the myogenic response to increasing blood pressure using laser‐doppler flowmetry in vitro. In the current study, we report no changes in dynamic autoregulatory capacity following −20 mmHg LBNP between placebo and fluconazole; however, there are large methodological differences between these investigations that may account for these discrepancies.

Beyond the already aforementioned differences between laser‐doppler flowmetry and transcranial doppler, the largest methodological difference between each study is the use of hyper‐ or hypotensive stimuli to stress the cerebrovasculature. Our investigation employed slight hypovolemic stress to induce a stage 1 hypovolemic response in the cerebrovasculature, characterized by a dilation of the vasculature to maintain blood delivery to the brain despite a reduction in blood pressure (Armstead, 2016). At −20 mmHg LBNP, this hypovolemic stimulus should be easily compensated by the body, restoring steady state blood pressure to regulated levels—as seen in our data by an increase in HR and a positive change in TPR, although not significant between the means. However, when analyzing dynamic autoregulation, it is important to remember that dynamic autoregulation analyses look for the rapid (dynamic) change in vascular responses to stress and thus includes the initial hypotensive stimulus as the start of LBNP. Gebremedhin's design utilized a hypertensive stimulus to activate an autoregulatory response, classically characterized as an increase in vasoconstriction to limit excessive pressure and blood volume on the brain's microvasculature (Armstead, 2016; Gebremedhin et al., 2000). In this way, each study is investigating a different component of the same mechanism and is not directly comparable to one another, as previous work has demonstrated that the human dynamic autoregulatory response may be better equipped to buffer rising (rather than falling) blood pressures (Tzeng et al., 2010). Thus, it is sensible that dynamic autoregulatory responses to hypovolemia, which would predominantly cause dilation, would not be like those found with hypertensive stimuli, which cause vasoconstriction in the brain's vasculature.

Moreover, this study implemented both time‐domain and frequency domain analyses to quantify dynamic autoregulatory changes, while previous investigations only utilized basic time‐domain methods (Gebremedhin et al., 2000). Utilizing the time‐domain assessment of AUC of the subcomponents of autoregulation (Beishon et al., 2018), our study was able to compare the relative changes in MCAv by subdividing its responses into metabolic, pressure, and myogenic contributions over the course of the entire duration of LBNP. In this way, we can expand on a similar premise set forth by Gebremedhin et al. (2000) but further give insight into the possible location of cerebrovascular control that is promoting these changes in velocity. As stated, our results indicate that CYP450 inhibition and placebo subcomponent responses are not different. However, each of these subcomponents responded in an expected manner to a lowering of blood pressure, where there is an increased contribution of myogenic activity, decreased contribution of metabolic activity, and little to no change in the contribution of blood pressure (Panerai et al., 2021).

Furthermore, transfer function analysis allows researchers to quantify low‐pass filter‐like effects of CA into comparable variables like gain (the change in signal amplitude), phase (the change in timing between signals), and coherence (the model's linear strength) (Panerai et al., 2023). Because of this, so long as the coherence within the low frequency band of a subject's data surpasses a set threshold, comparisons between gain and phase changes can be performed. Moreover, during resting conditions where no forced oscillatory stimuli is occurring to artificially increase data coherence, researchers have been able to interpret low coherence values as intact autoregulatory control (Claassen et al., 2021). Our data showed that 13 of our participants had a coherence below the determined threshold of 0.34, signifying that most participants retained healthy autoregulatory capacity even after treatment. Those that did have their gain and phase compared showed no effect of time, treatment, or a time × treatment interaction, although low frequency phase and gain did show moderate to large interaction effects. This is likely due to the low sample of participants that were able to be analyzed. Due to this, it is possible CYP450 inhibition affects dynamic autoregulatory phase and gain and that the current study is unable to properly power the analysis. This warrants further investigation.

### 4.3 Limitations and future considerations

This study is not without methodological limitations. First, although nonsignificant, MCAv, CVCi, RAP, CrCP, phase, and gain all exhibited moderate‐to‐large interaction effect sizes—indicating a lack of statistical power to see true effects of the FLZ treatment. To determine this, we calculated both achieved power and a priori power to determine the sample size that would be needed for MCAv to achieve proper power (*β* = 0.85) based on our observed effect size ($\eta_{p}^{2}$ = 0.026). We found that we achieved a power of 0.41, where a sample size of 60 subjects would be required to reach a power of 0.85. This was substantially larger than the initial estimation of 14 subjects originally needed and demonstrates the more subtle, likely redundant effects of CYP450 on the vasculature. Although underpowered, it is important to remember that *p* values are not the sole indicator of treatment effectiveness in medical and clinical research (Miola & Miot, 2021). The current study not only demonstrated substantial effect sizes across different cerebrovascular outcomes but also showed within‐treatment effects only within FLZ utilizing highly conservative Bonferroni post hoc tests. Paired together, these moderate‐to‐large interaction effects, significant and large main effect of time, with conservative post hoc testing suggest an effect of CYP450 on cerebral blood hemodynamics, although a nonsignificant interaction was observed. However, these results should be viewed as exploratory and interpreted cautiously due to the lack of power in the current study, the indirect nature of this conclusion, and high variation seen in the data. More research is needed to solidify this possibility.

Studies examining the role of CYP450 in the human periphery have demonstrated these enzymes likely play a redundant role in the vasculature and become more prominent in times of endothelial dysfunction. Ozkor et al. (2010) observed reduced forearm blood flow in healthy, young adults following intravenous administration of fluconazole only following nitric oxide blockade and within diabetic participants. There was no change in vascular responses in healthy participants. This is likely due to the inhibitory effect of NO on CYP450 activity, which limits CYP450 epoxygenase activity when NO synthesis is high (Stadler et al., 1994). Thus, it is likely that the use of healthy individuals in the current study limited CYP450 activity and reduced its observable effects.

Additionally, due to this redundancy, it is likely that −20 mmHg LBNP in healthy, young adults was not a strong enough pressure stimulus to induce CYP450's redundant mechanisms in the cerebrovasculature. Our choice to use −20 mmHg ensured that systemic blood pressure alterations would not affect static hemodynamic regulation regardless of individual sensitivity to lower body negative pressure (i.e., every participant would be able to properly regulate blood pressure during LBNP); however, by doing so, we likely did not select a strong enough stimulus to force a more active need for this pathway. These items could explain the required large sample size to properly power the redundant effects of these enzymes on cerebral hemodynamics in healthy, young adults—which is already comprised of numerous other redundant mechanisms.

Given the variable nature of neuronal and the likely decline of endothelial‐dependent NO bioavailability with aging and disease (Domek‐Łopacińska & Strosznajder, 2010), as well as the delicate tradeoff between recruiting a strong stimuli while maintaining vascular activity in isolation of systemic pressure changes, future studies should investigate CYP450's role in cerebral hemodynamics in populations with higher risk of NO depletion where cytochrome P450 activity would be greatest and possibly play a more necessary role in hemodynamic control during −20 mmHg of LBNP. For future studies involving healthy, young participants, we would recommend using a pressure greater than −20 mmHg, but no greater than −40 mmHg—as studies have demonstrated that −40 mmHg is powerful enough to alter vascular resistance of the MCA while not affecting diameter, indicating strong arteriole and capillary activation (Serrador et al., 2000). Moreover, −40 mmHg appears to be the largest negative pressure, on average, that most humans can properly compensate for to maintain blood pressure—thus limiting the confounding effects of blood pressure on velocity (Rickards, 2015). It is also recommended, to help lower individual variation in the response to LBNP, to employ a relative negative pressure determined by each subject's LBNP tolerance. For dynamic analysis of autoregulation, we would additionally recommend adding a separate stage of oscillatory LBNP to enhance the signal‐to‐noise ratio in the low frequency range for participants. By doing this, researchers can increase participants' coherence to an acceptable level to be included in transfer function analysis—increasing the statistical power and reliability of gain and phase results (Smirl et al., 2015).

Second, the current design only reflects the role of CYP450 in cerebral hemodynamics in healthy, young adults and cannot be generalized to clinical or aging populations. Next, the use of orally administered fluconazole likely resulted in the inhibition of CYP450 systemically, including within neurons and glial cells due to the drug's ability to pass through the blood brain barrier (Grant & Clissold, 1990). Because of this, the current investigation is unable to make claims to the exact location (endothelium, neurons, astrocytes, and vascular smooth muscle) where any observed effects occurred and can only report the summed effects on cerebral hemodynamics. Additionally, although fluconazole has excellent absorption into the plasma, the choice of a singular oral dose likely diminished the potency of fluconazole compared to acute local infusion (Grant & Clissold, 1990). We unfortunately did not measure peak plasma concentrations of fluconazole during the study, but previous pharmacokinetic work has demonstrated that 2 h is ideal for reaching peak plasma concentration (Grant & Clissold, 1990). Still, we would recommend measuring plasma levels to ensure peak concentrations were met for future studies. Due to the safety and practicality of local drug administration to the brain, we chose to administer an oral dose to participants.

Furthermore, the use of current equipment, such as transcranial doppler, hosts in own limitations. While excellent with temporal measures, TCD lacks the ability to image the arteries of the brain and thus requires the assumption that the diameter of the vessel must remain constant. Evidence has shown that this is not always the case, specifically in incidences of CO_2_ rebreathing that cause a change in at least 7.5 mmHg PETCO2 (Verbree et al., 2014). However, MRI studies investigating MCA diameter during −20 mmHg LBNP—the same strength as our own stimulus—has demonstrated no change in diameter (Serrador et al., 2000). Thus, it is unlikely that MCA diameter was changed following LBNP activation or by minor fluctuations in PETCO2 seen in our subjects, making our TCD measures reliable and valid. Further, near infrared spectroscopy is also affected by the thickness of subcutaneous fat (Niwayama et al., 1999) and we did not do any corrections for this fat layer. However, given our sample population was nonobese and TSI was measured on the forehead—we do not believe that this extremely thin fat layer would present any complications for measurement or data interpretation.

This study did not experimentally control PETCO_2_ and demonstrated a significant reduction in end‐tidal carbon dioxide only within the control condition. Further, research has shown that even a small amount of change in arterial CO_2_ could affect flow through a vessel (~1%–3% per 1 mmHg decrease in arterial CO_2_) (Claassen et al., 2021). To account for this, we utilized a mixed‐effects model and demonstrated the effect of PETCO_2_ on cerebrovascular variables. We found that PETCO_2_ presented no effect on MCAv, RAP, TSI, or CVCi. We did observe the effect of PETCO_2_ on CrCP. This is unsurprising given the strong relationship between critical closing pressure and CO_2_ (Panerai, 2003). Unfortunately, due to PETCO_2_ only decreasing in placebo condition, results of CrCP in our data become more difficult to discern. Of interest, we saw that CrCP continued to decrease despite falling PETCO_2_ levels within PLC. This observation is directly opposing typical responses seen during altered blood CO_2_ in other studies (Panerai, 2003). This likely reflects strong vasodilatory mechanisms that are overriding the vasoconstrictive response to depleting end tidal CO_2_ in the blood, highlighting the priority and importance of increased conductance in the microvasculature to maintain flow in the face of hypovolemic stress.

Additionally, the lack of dilation in FLZ without an additive challenge of CO_2_‐dependent constriction hints that this urgent, overpowering dilatory effect is likely removed with the attenuation of CYP450 epoxygenase activity. This could suggest that, during hypovolemic stress, CYP450 activity could help maintain microvascular dilation when other accumulative constrictive signals may seek to counteract this needed reduction in CrCP. More research is needed on the cerebrovascular responses during hypovolemia with hypocapnia to clarify this potential mechanism. Overall, given the strong modulatory effects of blood gas levels on the cerebrovasculature, future research should consider experimentally controlling this through controlled breathing or by CO_2_‐clamp methods.

## 5 CONCLUSIONS

At the time of writing, this study is the first to examine CYP450's role on cerebral hemodynamics in human participants. Our results, while statistically insignificant, present subtle evidence for CYP450's role in the human cerebrovasculature. However, the current study is statistically underpowered to generate a statistically significant *p* value, despite the moderate‐to‐large observed effects of CYP450 inhibition on cerebrovascular control and should be interpreted cautiously. Moreover, our findings indicate no differences in hemodynamic responses between the sexes following CYP450 blockade. Future research should explore this mechanism in those with known endothelial dysfunction and altered nitric oxide bioavailability (e.g., aging, stroke, hypertension, etc.) and expand on possible sex‐dependent mechanisms between these states.

## AUTHOR CONTRIBUTIONS

J.M.K, J.E.M, A.A.B, C.D.B, and H.M.P conceived and designed research; J.E.M, A.A.B, S.M.S., J.D.A, C.M, J.S, and A.A conducted and performed experiments; J.E.M, J.M.K, P.P, J.D.S, and C.M analyzed data; J.E.M and J.M.K interpreted results; J.E.M and A.A.B prepared figures; J.E.M drafted manuscript; All authors edited and approved final version of manuscript and ensured the accuracy and integrity of the work. All who qualify for authorship have been listed.

## FUNDING INFORMATION

No funding was received for this work.

## CONFLICT OF INTEREST STATEMENT

No conflicts of interest, financial or otherwise, are declared by the authors.

## Data Availability

Data is available upon request.
